# Respiratory symptom burden, vaccination coverage, and preventive health practices among Sudanese Hajj pilgrims who traveled by sea

**DOI:** 10.3389/fpubh.2025.1702386

**Published:** 2025-11-24

**Authors:** Najim Z. Alshahrani, Mohammed R. Algethami, Abdulrahman M. Albeshry, Zuhier Awan, Wael Alzhrani, Bashaier Ahmed Fairaq, Harunor Rashid

**Affiliations:** 1 Department of Family and Community Medicine, College of Medicine, University of Jeddah, Jeddah, Saudi Arabia; 2 College of Applied Medical Sciences, University of Jeddah, Jeddah, Saudi Arabia; 3 Population Health Management, Jeddah First Health Cluster, Jeddah, Saudi Arabia; 4 Population Health Management, Makkah Health Cluster, Makkah, Saudi Arabia; 5 Children’s Hospital Westmead Clinical School and Sydney Infectious Diseases Institute, University of Sydney, Westmead, NSW, Australia

**Keywords:** Hajj, preventive health practices, respiratory symptoms, Sudanese pilgrims, vaccination, influenza, mass gathering, sea travel

## Abstract

**Background:**

The risk of respiratory infections amplifies at Hajj. Pilgrims who travel by sea may face an elevated risk of such infections; however, contemporary Hajj literature offers limited data on this subgroup. This study assessed respiratory symptom burden, vaccination coverage, and preventive practices among Sudanese pilgrims who traveled to the 2025 Hajj by sea.

**Methods:**

A descriptive cross-sectional survey was conducted among Sudanese Hajj pilgrims who reached Saudi Arabia by sea. Data were collected using a structured, pilot-tested questionnaire covering demographics, medical history, preventive practices, awareness, and symptom experiences. Statistical analysis included descriptive measures, chi-square tests, correlations, and logistic regression to identify predictors of adherence to health recommendations.

**Results:**

A total of 370 pilgrims aged 25 to 87 years were recruited, comprising 114 (30.8%) men and 256 (69.2%) women. About 60.5% reported at least one health symptom, with muscle/body aches, cough and sore throat being the leading complaints. Symptom burden was significantly higher among older pilgrims, males, individuals with chronic conditions, and those with prior Hajj experience (*p* < 0.001 for all). Vaccine uptake was high for mandatory vaccines: meningococcal ACWY (95.1%), influenza (90.5%), and yellow fever (90.5%), but low for non-mandatory vaccines such as polio (8.6%) and hepatitis B (7.0%). A high level of confidence in the Saudi healthcare system (94%), high awareness of risks (86.0%), pre-travel preparedness (83.2%), frequent hand washing (55.9%) and confidence in prevention (89.5%) were strongly associated with adherence to recommended practices.

**Conclusion:**

These findings suggest that Sudanese pilgrims who travel by sea generally comply with mandatory preventive measures, but show lower adherence to non-mandatory ones, leaving them vulnerable to infections. Strengthening structured pre-travel health education and ensuring equitable access to vaccinations could significantly improve their health outcomes.

## Background

Hajj pilgrimage is one of the largest annual mass gatherings in the world, drawing millions of Muslims from diverse countries into a confined geographic area over a short period (1). The dense congregation of people, combined with intense physical exertion, extreme climatic conditions, and extensive international travel, creates an environment conducive to the spread of infectious diseases (2). These factors make Hajj a setting of unique public health significance, necessitating meticulous preparation, preventive strategies, and evidence-based risk management (3).

In collaboration with international health organizations, the Saudi government has implemented extensive healthcare, infrastructure, and emergency response systems to address these challenges (4, 5). Mobile clinics, temporary hospitals, and coordinated public health campaigns aim to detect, treat, and prevent illnesses during the event, while ongoing adaptations ensure responsiveness to the dynamic conditions of the pilgrimage environment (6, 7).

Sudan, as a participant nation in Hajj, presents a particular epidemiological profile that warrants focused attention. The country continues to face a considerable burden of communicable diseases, including tuberculosis, malaria, and other endemic infections, alongside a rising prevalence of non-communicable conditions such as diabetes, hypertension, and cardiovascular disorders (8, 9). These health challenges are further influenced by variable healthcare infrastructure capacity and disparities in access to preventive and curative services amidst ongoing conflicts (5).

When Sudanese pilgrims join Hajj, their health status and risk exposures intersect with the unique environmental, logistical, and cultural dynamics of the pilgrimage (8). This intersection heightens both their vulnerability to adverse health outcomes and the potential for bidirectional transmission of infectious agents into Saudi Arabia during the event and back into Sudan or other countries on return (10). These concerns extend beyond individual well-being to encompass broader regional and global health security (11).

Despite the scale of these risks, research specifically examining the health vulnerabilities, preventive practices, and behavioral determinants among Sudanese pilgrims remains scarce. Each year over 12,000 devotees make pilgrimage to Mecca from Sudan by air and sea, but no focused study has explored the health behaviors of these pilgrims. Most of the literature to date has focused on either multinational pilgrim cohorts or travelers from higher-income settings, which may not reflect the sociocultural contexts, health system realities, or behavioral patterns of Sudanese participants (12). Specifically, data on Sudanese pilgrims who attend Hajj via sea route are non-existent. Historical studies from the last century, when sea travel was a common mode of transport to Mecca, revealed that these voyages were frequently associated with major epidemics such as cholera, smallpox, and plague. Although a few recent surveillance studies have included Hajj pilgrims who traveled by sea, none have specifically focused on Sudanese pilgrims using this route (13, 14).

Consequently, there is a critical need for context-specific evidence to inform targeted health education, risk reduction strategies, and policy interventions that are both culturally and logistically appropriate for this group. The present study addresses this gap by conducting a comprehensive cross-sectional assessment of health risks and preventive strategies among Sudanese pilgrims traveling by sea during the 2025 Hajj.

## Methods

### Study design, setting, and population

A cross-sectional survey was conducted among Sudanese pilgrims who traveled by sea and entered Saudi Arabia via Jeddah Islamic Port, a major maritime hub for Hajj and Umrah pilgrims, including those from Sudan. Data collection was carried out from May 21 to June 18, 2025 (corresponding to 15 days before Hajj to 10 days after Hajj). Surveys were administered in designated passenger-processing areas of the port, including arrival halls, waiting lounges, transport staging zones, and departure terminals. Conducting the study in this single, standardized setting ensured consistency of procedures and facilitated a reliable assessment of health behaviors, perceptions, and outcomes during the pilgrimage.

### Inclusion and exclusion criteria

Eligible participants were Sudanese male and female pilgrims aged 18 years and older who provided informed consent and were able to communicate their responses. Pregnant and post-partum women as well as individuals who could not provide consent were excluded from participation.

### Sample size calculation

Approximately 11,500 Sudanese pilgrims participated in Hajj during the study period, of whom around 5,000 traveled to Saudi Arabia by sea (15). The minimum sample size required was determined using the Raosoft online calculator (16). The calculation incorporated a 95% confidence level, an assumed response proportion of 50%, and a margin of error set at 5%. Based on these parameters, the estimated sample necessary to ensure adequate statistical power was 367 participants. To account for an anticipated 10% non-response rate (90% expected response rate), approximately 410 questionnaires were distributed during the data collection period, of which 370 were completed and included in the final analysis.

### Questionnaire design and data collection

Data were collected using a structured, anonymous questionnaire in Arabic language developed specifically for this study. The instrument was informed by existing literature and previously validated health behavior assessment tools to ensure comprehensive coverage of relevant domains (17–20). The questionnaire consisted of five major sections: (i) demographic characteristics (age, gender, educational attainment, prior Hajj experience); (ii) medical history, including the presence of chronic conditions such as cardiovascular disease, diabetes, and respiratory disorders; (iii) preventive health measures, such as vaccination history, adherence to hygiene guidelines, dietary practices, and use of protective equipment; (iv) awareness of health risks, confidence in self-protection, self-reported symptom experience during Hajj, healthcare service utilization, and trust in official communications; and (v) cultural influences on behavior, perceptions of other pilgrims’ practices, readiness for health challenges, and engagement with digital health tools.

In addition, participants were asked to identify what they perceived to be the most significant overall health challenge for pilgrims during Hajj (infectious diseases, non-communicable diseases, environmental factors, psychological stress, or other). This measure captured pilgrims’ perceptions of health risks affecting the wider pilgrim community, rather than their own symptoms.

Content validity of the draft questionnaire was evaluated by a multidisciplinary panel of four experts: two professors of preventive medicine and public health with expertise in mass-gathering medicine; one consultant infectious-diseases physician with field experience in Hajj health services; and one senior epidemiologist with expertise in survey design and psychometrics. Each expert independently assessed item clarity, relevance, comprehensiveness, and sequencing; discrepancies were resolved by consensus. The panel’s feedback informed refinements to item wording, response options, and overall structural coherence of the instrument.

The content validity index (CVI) was determined at three levels: item (I-CVI), expert (E-CVI), and scale (S-CVI) (21). At the item level, the I-CVI was calculated as the proportion of experts rating an item as relevant or clear (rating ≥3) out of the total number of experts. The expert-level CVI (E-CVI) was calculated as the proportion of items rated as relevant by each expert. Items were considered appropriate if I-CVI was >0.79, required revision if between 0.70 and 0.79, and were deleted if <0.70. The scale-level CVI (S-CVI) was obtained by averaging the I-CVI values across all items, with an S-CVI ≥ 0.90 indicating excellent overall content validity.

Following expert review, a pilot test was conducted with 30 randomly selected Sudanese pilgrims who arrived in Saudi Arabia approximately 16 days before the start of the Hajj 2025. These individuals had traveled from Sudan by sea and were preparing to participate in the pilgrimage. The pilot study was implemented in the initial days of pilgrim arrivals to allow sufficient time for rapid refinement of the instrument. The research team immediately analyzed pilot data and incorporated necessary corrections on the same day to ensure timely finalization of the questionnaire. The mean completion time was 10–15 min, and no major difficulties in comprehension were reported. Internal consistency reliability was assessed using Cronbach’s alpha, which yielded a coefficient of 0.71, indicating acceptable reliability for large-scale field implementation.

### Data collection approach

Data were collected through face-to-face administration of the Arabic-language questionnaire by a team of 10 trained research assistants at Jeddah Islamic Port. Pilgrims were approached in designated waiting areas, including arrival halls, lounges, and departure terminals, to minimize disruption to port operations. After providing informed consent, participants completed the survey under the supervision of the research team, who were available to clarify any questions. Each participant was surveyed once during the study period, which spanned the Hajj season, ensuring representation across different phases of travel. This approach facilitated direct engagement, reduced the likelihood of missing data, and ensured consistency in administration procedures.

### Statistical analysis

Data were entered and cleaned in Microsoft Excel and analyzed using IBM SPSS Statistics, Version 26 (IBM Corp., Armonk, NY, United States). Descriptive statistics summarized participant characteristics, preventive practices, awareness, and health experiences. Categorical variables were reported as frequencies and percentages, and continuous variables as means with standard deviations (SDs) or medians with ranges. Associations between socio-demographic factors and self-reported health symptoms were assessed using Pearson’s chi-square tests, while correlations between pre-travel preparedness, awareness, and behavioral indicators were examined with Pearson’s correlation coefficients. Logistic regression was applied to identify independent predictors of adherence to health recommendations, with results presented as adjusted odds ratios (aOR) and 95% confidence intervals (CIs). The logistic regression model was adjusted for key sociodemographic variables, namely age, gender, and presence of chronic diseases. Only fully completed questionnaires were included in the final analysis. Partially completed surveys or those with missing responses were excluded to ensure data integrity and consistency across all variables. No data imputation or substitution was performed. Statistical significance was set at *p* < 0.05.

### Ethics approval and consent to participate

Ethical approval for this study was obtained from the Research Ethics Committee at the Ministry of Health, Jeddah, Saudi Arabia, Approval No. (A02212). The study was conducted in accordance with the Declaration of Helsinki principles and national research ethics guidelines. All participants were provided with detailed information about the study objectives and procedures through an electronic information sheet before accessing the survey. Informed consent was obtained electronically from all participants before they could proceed with the questionnaire. Participation was entirely voluntary, and participants could withdraw from the study at any time before submitting their responses. No personal identifying information was collected to ensure anonymity. The collected data were used solely for research purposes, and participants were assured that their responses would be analyzed and reported only in aggregate form.

## Results

### Demographic characteristics and symptom experience during Hajj

A total of 370 participants completed the survey. The mean age of the participants was 53.1 years (SD = 10.5; range: 25–87 years). Table 1 outlines the demographic profile of Sudanese pilgrims and its relationship with the occurrence of health symptoms during Hajj. Most participants were aged over 50 years (60.1%), and this group reported a significantly higher prevalence of symptoms compared with younger pilgrims (68.0% vs. 49.7%, *p* < 0.001). Women comprised the majority of the cohort (69.2%) and experienced symptoms less frequently than men (54.7% vs. 73.7%, *p* < 0.001). The presence of chronic disease was strongly associated with symptom reporting, with affected pilgrims showing a markedly higher prevalence than those without chronic conditions (69.5% vs. 47.0%, *p* < 0.001). Similarly, having performed Hajj previously was linked to a greater likelihood of experiencing symptoms (67.4% vs. 58.4%, *p* < 0.001).

**Table 1:** Demographic characteristics of Sudanese pilgrims and their association with the presence of respiratory symptoms during Hajj.

Characteristic	Category	Total *n* (column %)	No symptoms *n* (%)	Experienced symptoms *n* (%)	*p*-value
Age group	≤50 years	151 (39.9)	76 (50.3)	75 (49.7)	<0.001*
	>50 years	219 (60.1)	70 (32.0)	149 (68.0)	
Gender	Male	114 (30.1)	30 (26.3)	84 (73.7)	<0.001*
	Female	256 (69.9)	116 (45.3)	140 (54.7)	
Chronic disease	No	166 (45.3)	88 (53.0)	78 (47.0)	<0.001*
	Yes	190 (51.9)	58 (30.5)	132 (69.5)	
Previous Hajj attendance	No	281 (76.7)	117 (41.6)	164 (58.4)	<0.001*
	Yes	89 (23.3)	29 (32.6)	60 (67.4)	

^*^Statistically significant *p*-value. *p* < 0.05 indicates statistical significance. n, number; %, percentage.

### Vaccine coverage among Sudanese pilgrims

**Figure 1:**
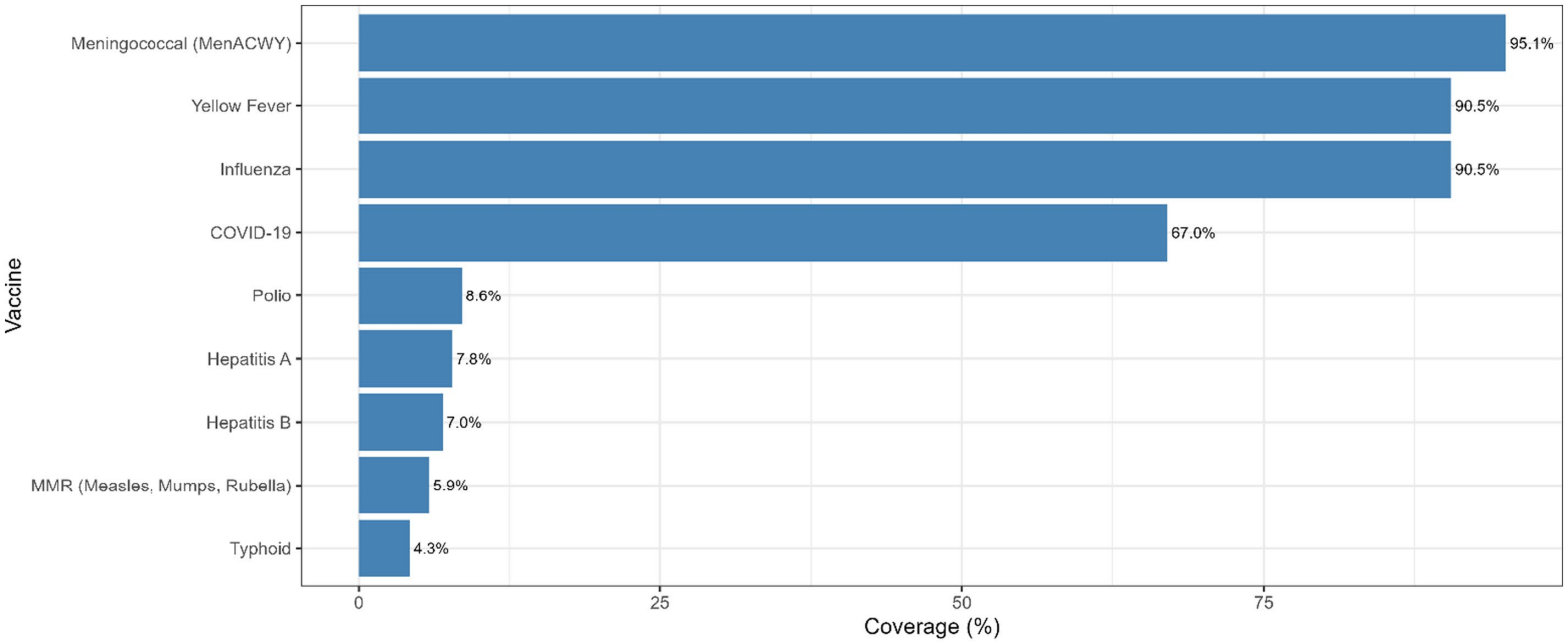
Vaccination coverage among Sudanese pilgrims.

Figure 1 illustrates vaccine coverage among Sudanese pilgrims. Uptake was highest for meningococcal ACWY (95.1%), followed closely by both influenza (90.5%) and yellow fever (90.5%). COVID-19 vaccination coverage reached 67.0%. Coverage for other vaccines was much lower, including polio (8.6%), hepatitis A (7.8%), hepatitis B (7.0%), measles, mumps and rubella (MMR) (5.9%), and typhoid (4.3%).

### Preventive practices, awareness, and symptom experience

As shown in Table 2, the majority of pilgrims demonstrated strong health awareness and pre-travel preparedness. Remaining well prepared to deal with health issues during Hajj was reported by 83.2% of participants, and 86.0% showed high awareness of potential health risks. Similarly, 89.5% expressed confidence in their ability to prevent health risks. Hand hygiene was widely practiced, with 55.9% reporting they “always” practiced handwashing or sanitizer use and another 33.8% reporting they did so “often.” Adherence to health recommendations was also strong, with 87.5% falling into the high adherence category. Awareness of healthcare services available during Hajj was common (80.0%), though only 30.5% reported using these services. Cultural background was perceived as a strong influence on health behaviors by 76.0% of participants, while almost all (94.3%) indicated no conflict between healthcare advice and cultural or personal beliefs. Use of digital tools to support health practices remained limited, with 74.2% reporting no use. Physical distancing was practiced frequently, with 66.5% reporting they did so “often” or “always.”

**Table 2:** Preventive practices, awareness, and symptom experience during Hajj.

Variable	Category	Column % (*n*)
a. Pre-travel preparedness, awareness, and confidence		
Preparedness to deal with health issues	Low (1–2)	4.1% (15)
	Moderate (3)	12.7% (47)
	High (4–5)	83.2% (308)
Awareness of potential health risks	Low (1–2)	3.7% (14)
	Moderate (3)	10.3% (38)
	High (4–5)	86.0% (318)
Confidence in preventing health risks	Not confident	1.1% (4)
	Somewhat confident	9.5% (35)
	Confident/Very confident	89.5% (331)
b. Preventive practices		
Handwashing/sanitiser use	Sometimes	10.3% (38)
	Often	33.8% (125)
	Always	55.9% (207)
Dietary guidelines	No special diet	41.6% (154)
	Mindful nutrition	31.4% (116)
	Special diabetic diet	12.4% (46)
	Vegetarian/Gluten-free/Lactose-free	8.6% (32)
	Other/Multiple diets	6.0% (22)
Adherence to health recommendations	Low (1–2)	2.2% (8)
	Moderate (3)	10.3% (38)
	High (4–5)	87.5% (324)
Physical distancing	Rarely/Never	11.6% (43)
	Sometimes	21.9% (81)
	Often/Always	66.5% (246)
c. Health services and cultural factors		
Awareness of healthcare services	Yes	80.0% (296)
	No/Not sure	20.0% (74)
Use of healthcare services	Yes	30.5% (113)
	No / Not sure	69.5% (257)
Cultural background influence	Low (1–2)	7.6% (27)
	Moderate (3)	16.4% (58)
	High (4–5)	76.0% (269)
Conflict with personal/cultural beliefs	No	94.3% (349)
	Yes	5.7% (21)
Use of digital tools for health practices	None	74.2% (271)
	Occasionally	17.6% (65)
	Regularly	8.1% (30)

n, number; %: percentage. Preparedness, awareness, confidence, and adherence were rated on a 5-point Likert scale (1 = lowest, 5 = highest).

### Self-reported health symptoms during Hajj

**Figure 2:**
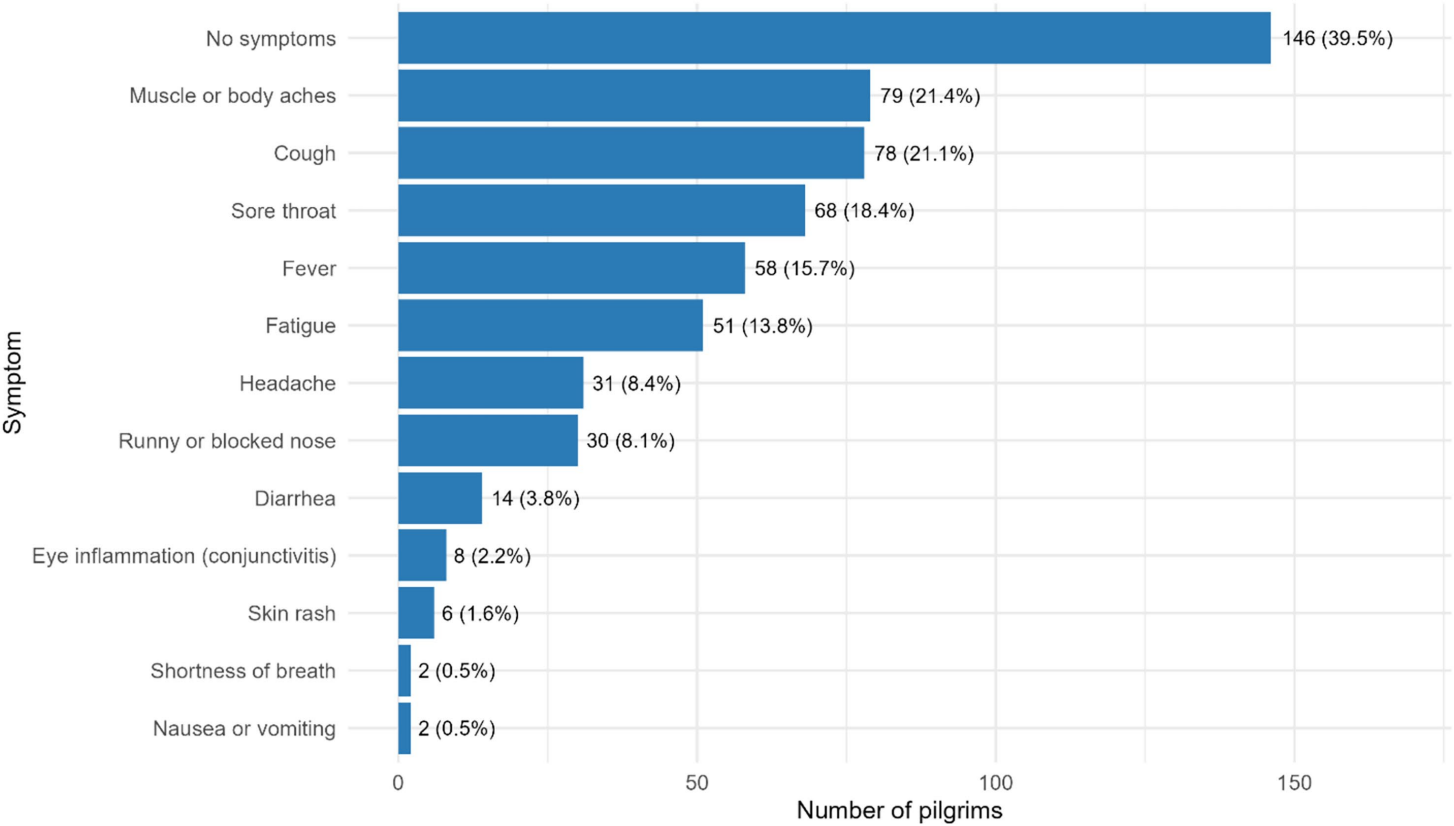
Self-reported respiratory symptoms experienced by Sudanese pilgrims during Hajj.

As presented in Figure 2, 39.5% of pilgrims reported no symptoms. The most frequently reported symptoms were muscle or body aches (21.4%), cough (21.1%), sore throat (18.4%), fever (15.7%), and fatigue (13.8%). Less common symptoms included headache (8.4%), runny or blocked nose (8.1%), and diarrhea (3.8%), while eye inflammation (2.2%), skin rash (1.6%), nausea or vomiting (0.5%), and shortness of breath (0.5%) were rarely reported. Many of these symptoms occurred in combination rather than in isolation.

### Health behaviors, perceptions, and experiences

As shown in Table 3, confidence in the Saudi healthcare system was very high, with 94.0% of participants rating it in the high category. The behavior of other pilgrims was generally perceived positively, with 60.6% reporting a positive influence on their own health practices. Most participants (87.0%) indicated an intention to participate in Hajj again based on their health experience. One-quarter (25.4%) sought medical care during Hajj, while healthcare services were rated highly by 89.7% of respondents. About 46.5% reported using traditional or home remedies, and 39.5% made lifestyle or health practice changes following Hajj. Cultural considerations in health guidelines were widely acknowledged, with 81.9% perceiving them as well integrated. However, most pilgrims (73.8%) reported not receiving a health kit, and only 30.3% had received specific Hajj-related advice from a healthcare provider prior to travel.

**Table 3:** Health behaviors, experiences, and perceptions during Hajj.

Variable	Category	% (*n*)
a. Confidence, perceptions, and intentions		
Confidence in Saudi healthcare system	Low (1–2)	1.0% (4)
	Moderate (3)	5.4% (20)
	High (4–5)	94.0% (348)
Impact of other pilgrims’ behavior	Positive effect (slight/strong)	60.6% (224)
	Negative effect	5.9% (22)
	No effect	33.5% (124)
Intention to participate in Hajj again	Yes	87.0% (322)
	No	13.0% (48)
b. Healthcare use and quality		
Sought medical care during Hajj	Yes	25.4% (94)
	No	74.6% (276)
Rating of healthcare service quality	Low (1–2)	1.6% (6)
	Moderate (3)	8.6% (32)
	High (4–5)	89.7% (332)
c. Health practices and cultural considerations		
Use of traditional/home remedies	Yes	46.5% (172)
	No	53.5% (198)
Changes to lifestyle/health practices post-Hajj	Yes	39.5% (146)
	No	60.5% (224)
Cultural diversity in health guidelines	Low (1–2)	4.4% (16)
	Moderate (3)	13.8% (51)
	High (4–5)	81.9% (303)
d. Health kits and pre-travel consultation		
Provision of health kits	None provided	73.8% (273)
	Basic kit	8.1% (30)
	Standard kit	10.0% (37)
	Comprehensive kit	8.1% (30)
Pre-travel healthcare consultation	No consultation	50.8% (188)
	General advice	18.9% (70)
	Specific Hajj-related advice	30.3% (112)

n, number; %: percentage. Ratings were based on a 5-point Likert scale (1 = lowest, 5 = highest).

### Most significant perceived health challenges during Hajj

**Figure 3:**
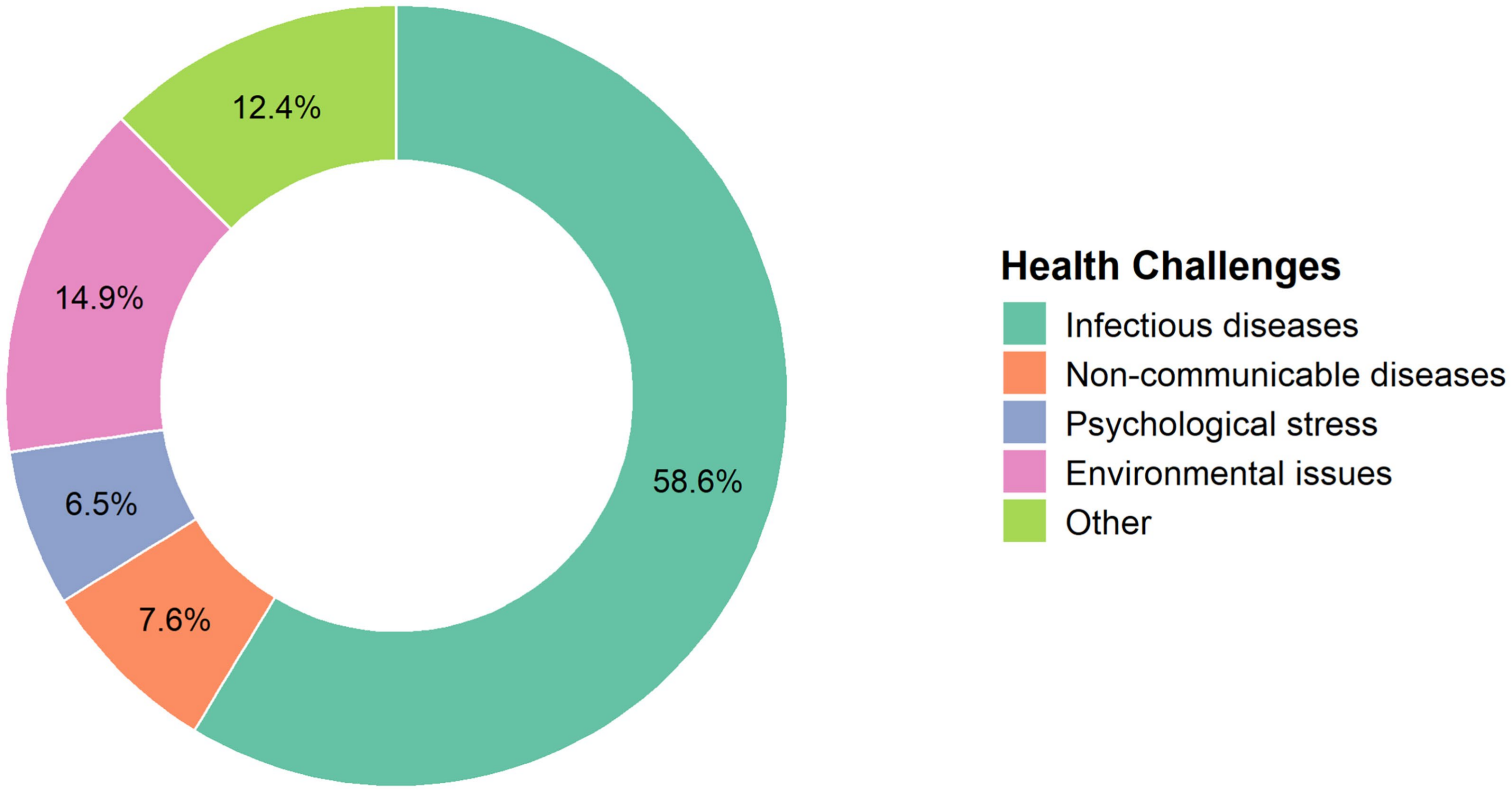
Main health challenges encountered by Sudanese pilgrims during Hajj.

Figure 3 illustrates the distribution of self-reported health challenges. This question was distinct from self-reported symptoms, as it reflected participants’ opinions about priority health risks to pilgrims as a group. Infectious diseases were the most frequently reported (58.6%), followed by environmental issues (14.9%) and other challenges (12.4%). Non-communicable diseases and psychological stress were reported less frequently (7.6 and 6.5%, respectively).

### Factors associated with adherence to health recommendations

Multivariable logistic regression identified several factors that were independently associated with higher adherence to health recommendations during Hajj (Table 4). Better preparedness to manage health issues (aOR = 2.0, 95% CI: 1.5–2.6, *p* < 0.001) and higher awareness of potential health risks (aOR = 2.4, 95% CI: 1.7–3.2, *p* < 0.001) were significant predictors of adherence. Pilgrims who reported strong confidence in their ability to prevent health risks were more likely to adhere (aOR = 7.3, 95% CI: 1.0–53.0, *p* = 0.049). Consistently practicing hand hygiene (“always” vs. reference) was also associated with higher adherence (aOR = 4.3, 95% CI: 1.6–11.5, *p* = 0.003). Additionally, higher confidence in the Saudi healthcare system was linked to increased adherence (aOR = 3.0, 95% CI: 1.8–5.01, *p* < 0.001).

**Table 4:** Factors significantly associated with adherence in logistic regression analysis.

Factor	Adjusted odds ratio (aOR)	95% CI	*p*-value
Pre-travel preparedness to deal with health issues (per unit increase)	2.03	1.56–2.63	<0.001*
Awareness of potential health risks	2.38	1.70–3.27	<0.001*
Confidence in ability to prevent health risks	7.32	1.01–53.00	0.049*
Frequency of hand washing/use of sanitizer (Ref = 0)			
Often	1.02	0.41–2.51	0.973
Always	4.33	1.62–11.58	0.003*
Confidence in the Saudi healthcare system	3.06	1.86–5.01	<0.001*

*Statistically significant *p*-value <0.05; aOR, adjusted odds ratio; CI, confidence interval; *p* < 0.05 indicates statistical significance.

### Recommendations to improve health communication and education

**Figure 4:**
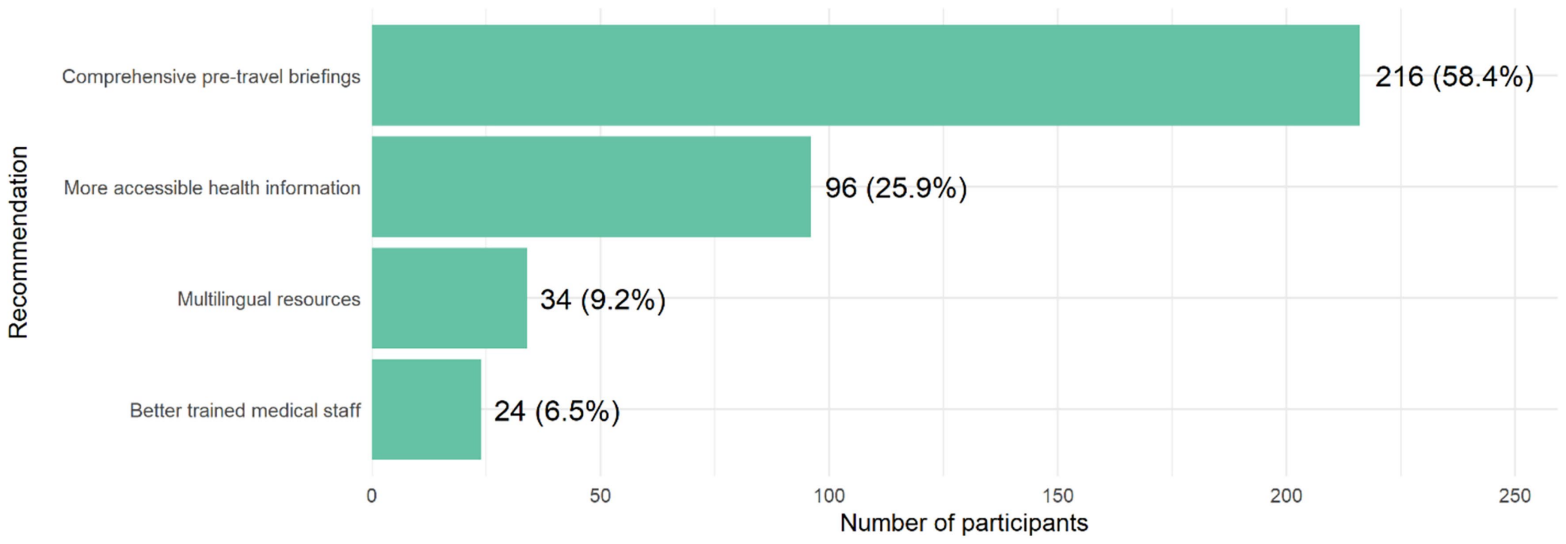
Pilgrims’ recommendations for improving health communication and education during Hajj.

Figure 4 presents recommendations provided by pilgrims. Comprehensive pre-travel briefings were the most common suggestion (58.4%), followed by making health information more accessible (25.9%), multilingual resources (9.2%), and better-trained medical staff (6.5%).

## Discussion

This study provides new evidence on the health experiences, preventive practices, and vaccination coverage of Sudanese pilgrims traveling by sea for the 2025 Hajj. Our findings show encouraging levels of health awareness and vaccine uptake, but also highlight ongoing challenges related to respiratory illness and overall symptom burden. These results add to the growing research on health risks in mass gatherings and have important implications for public health preparedness.

In this study, >60% of pilgrims reported at least one symptom during Hajj: notably muscle or body aches, cough, sore throat, fever, and fatigue. The high rate of musculoskeletal complaints likely reflects the physical strain of prolonged walking, standing, and ritual activities during Hajj, which can affect comfort and functional capacity (22, 23). Contextual factors such as long sea voyage, crowding, and seasonality may also have influenced the symptom profile observed. The 2025 Hajj occurred during a particularly hot and humid season. Prolonged exposure to high temperatures and physical exertion may have increased the risk of dehydration, and consequently musculoskeletal strain (24). Sea travel from Sudan to Jeddah typically takes several days under a crowded and semi-closed environment; limited mobility, poor sleeping arrangements, and restricted hygiene facilities aggravate musculoskeletal discomfort and facilitate pathogen spread. Previous studies found that sea and overland pilgrims face greater fatigue and respiratory risk than air travelers (23–25).

Respiratory symptoms accounted for a large share of the overall symptom burden, consistent with findings from other Hajj studies (24). Yezli et al. (25) found that 45% of clinic diagnoses during the Hajj 2019 were respiratory in nature, while Mahomed et al. (26) reported that 71% of South African pilgrims experienced respiratory symptoms, both during and after the pilgrimage. Collectively, these findings reinforce that respiratory illness remains the most prevalent health issue during Hajj, including among Sudanese pilgrims (26).

Despite the high prevalence of symptoms, illness severity was generally mild. Only one-quarter of participants sought medical care during Hajj. This supports previous evidence that most Hajj-related illnesses are self-limiting, though still disruptive to wellbeing and daily functioning. The clustering of symptoms observed in our data suggests a combination of viral and bacterial etiologies, consistent with findings from earlier microbiological studies (27, 28).

Vaccination patterns in our cohort were notable for very high uptake of the required meningococcal ACWY, yellow fever, and influenza vaccines, alongside more modest coverage for other vaccines. The meningococcal ACWY vaccine deserves particular emphasis, as it is a strict requirement for Hajj visa issuance, with Saudi regulations mandating documented proof of vaccination for every incoming pilgrim (29–31). In principle, this policy should guarantee 100% coverage. The slightly lower figure observed in our study (95.1%) is most likely attributable to reporting errors (31), and underscores the importance of validation through official health records in future studies (32). Nevertheless, the meningococcal ACWY vaccination rate as low as 79% was reported among international Hajj and Umrah pilgrims in 2024 (33).

Despite the encouraging levels of compliance with mandatory vaccines, the uptake of non-mandatory vaccines such as polio, hepatitis B, and typhoid remained notably low. Several factors may have contributed to this pattern: (i) these vaccines are not required for visa issuance or travel clearance, (ii), limited awareness among pilgrims, (iii) financial barriers (18), and (iv) logistical challenges, including access to vaccination in remote areas (33). To address these gaps, targeted interventions are required to enhance coverage for optional but beneficial vaccines.

The high coverage of influenza vaccination (90.5%) in our group is striking, especially when compared with the findings of Mahomed et al. (26) who found influenza vaccine uptake to be only 44% among South African pilgrims. Such differences may reflect successful public health campaigns targeting Sudanese pilgrims, or stronger integration of influenza vaccination into pre-departure requirements. Even with high coverage, however, respiratory symptoms remained common, underscoring that diverse respiratory pathogens, not just influenza and SARS-CoV-2, circulate at Hajj (24–27, 34). Promisingly, a cross-sectional study conducted in 2025 based on electronic health records from hospitals and primary health centers in Hajj spots in Makkah showed that vaccinated Sudanese pilgrims had lower incidence of influenza-like illness (ILI) compared to unvaccinated pilgrims (5.2% vs. 15.2%, *p* < 0.01) (35).

COVID-19 vaccination coverage in our cohort (67%) was notably lower than uptake for influenza and meningococcal vaccines, potentially reflecting pandemic fatigue or diminished perception of COVID-19 risk (35). In contrast, Alshamrani et al. (36) reported that nearly 90% of Saudi pilgrims had completed COVID-19 vaccination, a third also received booster doses. This disparity may be related to accessibility, awareness, or cost factors among Sudanese pilgrims, many of whom are older and likely to be of far less salubrious origin (35).

Preventive practices, particularly hand hygiene, showed strong adherence among study participants. Nearly 90% of them reported frequent or consistent handwashing, which is higher than has been described in some previous cohorts (37). Hand hygiene was also independently associated with adherence to broader health recommendations in our regression analysis, highlighting its central role in protective behavior (38–40). Nevertheless, the uptake of digital health tools was limited, suggesting an opportunity to modernize communication and education strategies for future pilgrimages. Emerging digital innovations, including artificial intelligence (AI), mobile health monitoring, predictive analytics, and automated triage systems could play a transformative role in strengthening health preparedness for future Hajj seasons (41, 42).

Another important finding was the very high level of confidence in the Saudi healthcare system (94%) and the strong perception of cultural alignment with health advice. This is encouraging, as perceived cultural compatibility can be a decisive factor in whether pilgrims accept or resist health recommendations (30). On the other hand, the limited receipt of health kits and relatively low rates of pre-travel medical counseling reflect gaps in the Sudanese pre-departure preparation process, rather than deficiencies in the services available in Saudi Arabia. Strengthening health education and counseling at the country-of-origin level would therefore be crucial to improving preparedness. Our participants themselves emphasized the importance of comprehensive pre-travel briefings followed by accessible health information, aligning with recommendations from Mahomed et al. (26) and Yezli et al. (25), who both highlighted the value of early intervention and targeted messaging in reducing illness burden, particularly in improving health communication and education during Hajj.

### Implications for public health and travel medicine policy

To our knowledge this is the first focused study to assess symptom burden and compliance with preventive practices among Sudanese Hajj pilgrims, and one of the few studies that involved pilgrims who reached Saudi Arabia via seaport. This study highlights important implications for both public health planning and travel medicine. The near-universal uptake of the mandatory meningococcal ACWY vaccine reflects the success of Saudi Arabia’s entry requirements, with the slightly lower reported figure likely due to recall or reporting issues rather than true gaps. In contrast, the much lower uptake of non-mandatory vaccines such as pneumococcal and hepatitis points to persistent gaps in voluntary preventive measures. Addressing these gaps will require stronger pre-travel counseling, targeted education, and closer collaboration between Saudi authorities, sending countries, and international health agencies. Finally, the strong links between pre-travel preparedness, awareness, and adherence underscore the need for culturally sensitive pre-departure health education to reduce illness burden and enhance pilgrim safety.

### Study limitations

This study should be interpreted in light of several limitations. The cross-sectional design limits causal inferences between individual characteristics, behavior, and health outcomes. Data were self-reported, raising the possibility of recall bias, reporting bias, and social desirability effects, particularly regarding preventive behaviors and vaccine history. The study was restricted to Sudanese pilgrims traveling by sea through Jeddah Islamic Port, which may limit generalizability to pilgrims from other countries or those arriving by air or land routes. Furthermore, the reliance on symptom self-reporting without laboratory confirmation restricts the ability to distinguish between viral and bacterial causes of respiratory illness. Another limitation concerns the wide confidence intervals observed in the logistic regression analysis for certain variables, e.g., pilgrims’ confidence in their ability to prevent health risks (OR = 7.32; 95% CI: 1.0–53.0). Despite the large effect size, the breadth of the interval suggests substantial variability within the sample and indicates a high degree of statistical uncertainty. However, this occurred only in a couple of variables, and the sample size was adequate. Finally, despite our attempts to have a balanced gender distribution, the data were skewed toward females, this could be because of women’s inherent eagerness to contribute to noble ventures including participating in a research survey. This gender imbalance may limit generalizability, as women’s health behaviors and risks may differ. Sensitivity analysis by gender was considered but not performed due to the small number of female participants. Future research should ensure more balanced representation to explore gender-specific health patterns and preventive practices. Despite these limitations, the study provides valuable insights into a relatively understudied population and setting, offering evidence that can inform targeted health interventions for sea-traveling pilgrims and contribute to broader mass-gathering health strategies.

## Conclusion

This study offers important insights into the preventive health practices of Sudanese pilgrims who traveled by sea to attend the 2025 Hajj. While uptake of mandatory vaccinations was nearly universal, adherence to optional vaccines remained suboptimal. Key factors such as pre-travel preparedness, health awareness, and confidence in healthcare system were strongly associated with adherence to preventive measures. These findings highlight the critical role of structured pre-travel health education and equitable access to vaccinations, particularly through targeted outreach to high-risk groups.

## Data Availability

The original contributions presented in the study are included in the article/supplementary material, further inquiries can be directed to the corresponding author.
